# Genomic analysis of *Salmonella enterica* from Metropolitan Manila abattoirs and markets reveals insights into circulating virulence and antimicrobial resistance genotypes

**DOI:** 10.3389/fmicb.2023.1304283

**Published:** 2024-01-19

**Authors:** Jonah Feliza B. Mora, Vanessa Yvonne B. Meclat, Alyzza Marie B. Calayag, Susana Campino, Julius C. R. Hafalla, Martin L. Hibberd, Jody E. Phelan, Taane G. Clark, Windell L. Rivera

**Affiliations:** ^1^Pathogen-Host-Environment Interactions Research Laboratory, Institute of Biology, College of Science, University of the Philippines Diliman, Quezon City, Philippines; ^2^Department of Infection Biology, London School of Hygiene and Tropical Medicine, London, United Kingdom; ^3^Department of Infectious Disease Epidemiology, London School of Hygiene and Tropical Medicine, London, United Kingdom

**Keywords:** *Salmonella enterica*, whole-genome sequencing, antimicrobial resistance genes, plasmids, virulence, food chains

## Abstract

The integration of next-generation sequencing into the identification and characterization of resistant and virulent strains as well as the routine surveillance of foodborne pathogens such as *Salmonella enterica* have not yet been accomplished in the Philippines. This study investigated the antimicrobial profiles, virulence, and susceptibility of the 105 *S. enterica* isolates from swine and chicken samples obtained from slaughterhouses and public wet markets in Metropolitan Manila using whole-genome sequence analysis. Four predominant serovars were identified in genotypic serotyping, namely, Infantis (26.7%), Anatum (19.1%), Rissen (18.1%), and London (13.3%). Phenotypic antimicrobial resistance (AMR) profiling revealed that 65% of the isolates were resistant to at least one antibiotic, 37% were multidrug resistant (MDR), and 57% were extended-spectrum β-lactamase producers. Bioinformatic analysis revealed that isolates had resistance genes and plasmids belonging to the *Col* and *Inc* plasmid families that confer resistance against tetracycline (64%), sulfonamide (56%), and streptomycin (56%). Further analyses revealed the presence of 155 virulence genes, 42 of which were serovar-specific. The virulence genes primarily code for host immune system modulators, iron acquisition enzyme complexes, host cell invasion proteins, as well as proteins that allow intracellular and intramacrophage survival. This study showed that virulent MDR *S. enterica* and several phenotypic and genotypic AMR patterns were present in the food chain. It serves as a foundation to understand the current AMR status in the Philippines food chain and to prompt the creation of preventative measures and efficient treatments against foodborne pathogens.

## 1 Introduction

Salmonellosis, caused by *Salmonella* spp., is among the most frequently reported foodborne diseases worldwide and has a high health and economic burden. *Salmonella enterica*, with its six distinct subspecies (I, II, IIIa, IIIb, IV, and VI), is a leading cause of global human diarrheal cases and outbreaks annually, including in the Philippines. While there are numerous potential means and sources of transmission, chicken and pig meat, along with other farm animals and products, have been identified as the dominant food vehicles for *S. enterica* due to their wide distribution and natural, chronic carriage among livestock (World Health Organization, 2018; Ferrari et al., 2019; Edrington and Brown, 2022). The predicted increase in consumption of swine and poultry products in the Philippines in the next ten years is over 3 million metric tons *per annum* (Organisation for Economic Cooperation and Development/Food and Agriculture Organization, 2023), which increases the potential for exposure to the pathogen. In 2021, the agricultural sector contributed to 9.6% of the Philippines’ national gross domestic product (GDP) (Philippine Statistics Authority, 2022). However, due to several outbreak events in the country such as African swine fever (Cooper et al., 2022) and COVID-19 pandemic (Espino et al., 2021), a significant decline in hog and poultry production output was reported. Approximately 16% (53 K metric tons) increase in imported frozen chicken meat was reported from 2020 to 2021 (Philippine Statistics Authority, 2022). In 2022 alone, pork (52%; 710 M metric tons) and chicken (30%; 411 M metric tons) topped the total meat importation to address the local supply shortage due to production loss and growing meat consumption (Bureau of Animal Industry, 2022).

Studies on raw and processed meats from abattoirs and wet markets in Metro Manila, Philippines, have revealed a high prevalence of *S. enterica* (>30%) and the additional high frequency of samples positive for the *spvC* virulence gene, which is strongly associated with strains that cause non-typhoidal bacteremia (Calayag et al., 2017; Santos et al., 2020). Subsequent work in Metro Manila has revealed highly frequent virulence genes (e.g., SPIs 1–5 genes), some co-occurring, and others linked to location and animal source (Pavon et al., 2022). In this setting, *S. enterica* has been identified primarily from chicken samples and ground pork (both >65%), and with multiple and mixed serogroups E1, C1, C2, B, and D being abundant (all >6%). *S. enterica* types vary significantly in their host range and their degree of host adaptation. Other studies set in Metro Manila in swine have found similarly high *S. enterica* bacterium prevalence across accredited and locally registered abattoirs (~50%) but with most bacteria under serogroup O (Ng and Rivera, 2015; Calayag et al., 2017). Collectively, these studies point to *S. enterica* circulating in the poultry and swine food chains in Metro Manila, with virulence genes, and thereby a likely major and increasing cause of gastroenteritis and enteric fever.

In 2015, a study in slaughtered swine in Metro Manila, Philippines detected five serotypes, namely, *S. enterica* Typhimurium, Agona, Heidelberg, Choleraesuis, and Weltevreden from tonsils and jejuna of freshly slaughtered swine (Ng and Rivera, 2015). In a separate work, serotypes Anatum, Kentucky, and Saintpaul have been found in bovine, porcine, and poultry meat from wet markets (Santos et al., 2020). The presence of these serotypes in meat samples can be correlated to disease. In a 15-year report of *Salmonella* serotype distribution in the Philippines by the Antimicrobial Resistance Surveillance Reference Laboratory of the Research Institute for Tropical Medicine (RITM), *S. enterica* Enteritidis, Typhimurium, Weltevreden, Stanley, and Anatum were found to be the five most prevalent non-typhoidal serotypes in clinical samples. The other serotypes found in meat samples such as Agona, Heidelberg, Choleraesuis, and Kentucky were also detected from clinical samples, although not as prevalent (Sia et al., 2020). This gives us a picture of how parallel the prevalent serotypes in meat samples and clinical samples are, and how tracking the source of these pathogens is paramount to reducing risk, designing mitigation strategies, and predicting future outbreaks.

Treatment options for salmonellosis are decreasing as the underlying bacteria continue to show antimicrobial resistance (AMR). Due to frequent antibiotic exposure, swine and poultry are now recognized as potential risks in disseminating drug-resistant *S. enterica*, with multidrug resistant (MDR) strains now being present in the Philippines (Calayag et al., 2017). As defined by Magiorakos et al. (2012), MDR organisms have non-susceptibility to at least one antimicrobial agent from three or more antimicrobial classes. Studies of *S. enterica* from slaughtered swine in Metro Manilla revealed high rates of resistance to ampicillin, trimethoprim/sulfamethoxazole, and MDR (all >67%) (Calayag et al., 2017). These forms of resistance can be detected by polymerase chain reaction (PCR)-based approaches that target known regions (e.g., on plasmids) or, phenotypically, using systems such as the VITEK® 2 Compact 60 ID/AST System (bioMérieux, 2005) for antimicrobial susceptibility testing. However, in other infection settings, e.g., tuberculosis (Phelan et al., 2019), *Klebsiella pneumoniae* (Spadar et al., 2023), next-generation sequencing (NGS) has gained traction for fast and affordable AMR profiling (genotyping). Whole-genome sequencing (WGS) analysis can be a rapid and cost-effective approach to define resistance genotypes, predict resistance phenotypes, and identify identical isolate genomes that are part of transmission chains (Sobkowiak et al., 2020; Napier et al., 2022).

To demonstrate the utility of sequencing, we have performed WGS on 105 *S. enterica* isolates across live animal and processed meat domains in the poultry and swine food chains in Metro Manila between 2018 and 2022. This study is part of a bigger project that aimed to recover 2,500 *S. enterica* isolates from abattoirs and wet markets in eight cities in Metro Manila, Philippines. The 105 isolates in this study have been obtained from six out of the eight cities, and were the first batch subjected to whole genome sequencing. The resulting genomic variation is used to understand circulating AMR and virulence gene repertoires. Our work provides a baseline set of genomic data and a snapshot of diversity, which can be used in emerging applications of NGS for the routine monitoring of meat product safety in the Philippines.

## 2 Materials and methods

### 2.1 Sample collection and processing

A collection of *S. enterica* strains isolated from swine and poultry meat was established to understand the AMR profiles and determine the virulence genes associated with swine and poultry food chains in Metro Manila, Philippines. The meat samples came from public wet markets and accredited as well as locally registered abattoirs in the four Metro Manila districts, namely, Capital, Eastern, Northern, and Southern. Sample collection from freshly slaughtered swine in slaughterhouses includes the 15-cm segment of the jejunum (Calayag et al., 2017). All sample collection was performed between 2018 and 2022. Different parts of swine and chicken meat were sampled from public wet markets. Raw meat samples include ground or cut-up meat, while processed meat samples include marinated, salted, cured, and pre-cooked products. The samples were transported to the laboratory in sterile plastic bags and kept cold in a cooler. Upon arrival at the laboratory, 25 g of meat sample was weighed and pre-enriched with 225 mL of sterile buffered peptone water (BD Difco, NJ, USA) in a sterile Rollbag® (Interscience, France), homogenized with BagMixer® 400 (Interscience, France) for 1 min, and incubated for 24 h at 37°C. For the single-enrichment broth culture method, 100-μL of the pre-enriched culture was transferred into Rappaport-Vassiliadis (RV) broth (10 mL; Difco, BD, Sparks, MD) and incubated at 42°C for 24 h (Ng and Rivera, 2015). A loopful of incubated RV broth was streak plated onto xylose lysine deoxycholate (XLD) agar (BD Diagnostics System, NJ, USA) plates for isolation and purification, and incubated at 37°C for 18–24 h. Typical *Salmonella* colonies, i.e., colonies with black centers and clear or transparent halo, were then subcultured on nutrient agar (NA) (BD Diagnostics System, NJ, USA) for further confirmation analysis (Pavon et al., 2022).

### 2.2 DNA extraction, molecular detection, and sequencing

Presumptive colonies of *S. enterica* were subjected to DNA extraction using a DNA purification kit (Monarch®, New England BioLabs, MA, USA) and stored in a −20°C freezer (Schnee Irish, Vienna, Austria). PCR-based identification was conducted using the species-specific *invA* gene to confirm the identity of the isolates (Ng and Rivera, 2015). The amplicons were subjected to agarose gel electrophoresis using 1.5% agarose stained with 10,000 × GelRed® in water (Biotium, CA, USA). Electrophoresis runs were made to proceed under 280 V for 35 min. The gels were visualized using a UV gel documentation system (Vilber Lourmat, France) (Pavon and Rivera, 2021). Amplicon size was estimated using 100-bp HyperLadder™ (Bioline, Meridian Bioscience, London, UK) as the molecular weight marker. Amplicons with the approximate size of ~244 bp were considered positive for the *invA* gene, and thus *Salmonella-*positive. The concentration and purity of the *invA*-positive DNA extracts were measured using a microplate spectrophotometer (Multiskan SkyHigh, Thermo Scientific™) before submission to the sequencing facilities. From a collection of 2,500 *S. enterica* isolates, a subset of 105 isolates was randomly selected for Illumina TruSeq library construction and sequencing undertaken at the DNA Sequencing Core Facility of the Philippine Genome Center and through the Applied Genome Centre at London School of Hygiene and Tropical Medicine (LSHTM).

### 2.3 AMR genotyping and phenotyping and virulence gene profiling

ABRicate software (v1.0.1; minimum %ID = 80, min % coverage = 80) (Seemann, 2020) was applied to find the presence of resistance genes (NCBI database, as of March 27, 2021) and plasmids (PlasmidFinder database; as of April 4, 2023) (Carattoli et al., 2014). The VITEK® 2 Compact 60 ID/AST System (AST-GN70 card panel, bioMérieux, Marcy-l’Étoile, France) was used to test the sensitivity of 105 isolates to 15 antimicrobial agents, including ampicillin, sulbactam, tazobactam, ceftriaxone, cefepime, aztreonam, ertapenem, meropenem, amikacin, gentamicin, tobramycin, ciprofloxacin, tigecycline, nitrofurantoin, and trimethoprim-sulfamethoxazole. Interpretive criteria and breakpoints from the Clinical and Laboratory Standards Institute (CLSI) (2022) 32nd Edition were used in the analysis. *S. enterica* subsp. *enterica* Le Minor and Popoff serovar Typhimurium ATCC 14028™, *Escherichia coli* ATCC 25922™, and *K. pneumoniae* ATCC 600703™ were used as quality control (QC) reference strains. ABRicate software (v1.0.1) (Seemann, 2020) with the same parameters was also applied to find the presence of virulence genes (VFDB database, as of April 4, 2023) (Chen et al., 2016).

### 2.4 Bioinformatics, sample genotyping, and phylogeny

Kraken2 software (v2.1.2) was used with the standard database to scan for any possible contamination (Wood et al., 2019). Genomes were assembled with Shovill software (v1.1.0) (Seemann, 2019) and were used by the SISTR tool (v1.1.1) (Yoshida et al., 2016) to predict serovars and cgMLST sequence types (STs), and to perform basic quality control checks. QUAST software (v5.2.0) was used to check the quality of the assemblies with all genomes passing a minimum of 95% of BUSCO genes found (Mikheenko et al., 2018). ParSNP software (v1.7.4) was used to perform a core genome analysis to identify genomic regions present in most strains. These regions were identified and aligned by ParSNP and this was used as input to build a phylogeny using the RAxML tool (v8.2.12; parameters: -m GTRGAMMA -N 100 -k -f a) (Stamatakis, 2014) for all isolates together, as well as for the four main individual serovar clades separately. The sequence data were scanned for 155 known virulence genes. The phylogenetic trees were used to identify potential transmission chains, through the application of established methods (Sobkowiak et al., 2020; Napier et al., 2022). The isolates have been run through Snippy (v4.6.0)^1^ (Seemann, 2015) to find single nucleotide polymorphisms (SNPs) and determine genetic similarities within serovars and across sampling sites (slaughterhouses and markets), cities, and matrices (swine or chicken). Genetically similar isolates (i.e., less than 20 SNPs) have been plotted in single-linkage clusters using transmission graph viewer.^2^

### 2.5 Ethical approval and consent to participate

Ethical review and approval were waived for this study due to informed consent obtained from the National Meat Inspection Service of the Philippine Department of Agriculture. Animal slaughter and evisceration were performed according to Philippine national regulations. Informed consent was also obtained from veterinarians in charge of the abattoirs, and farm owners for sample collection.

## 3 Results

### 3.1 Characteristics of the *Salmonella* isolates

**Figure 1:**
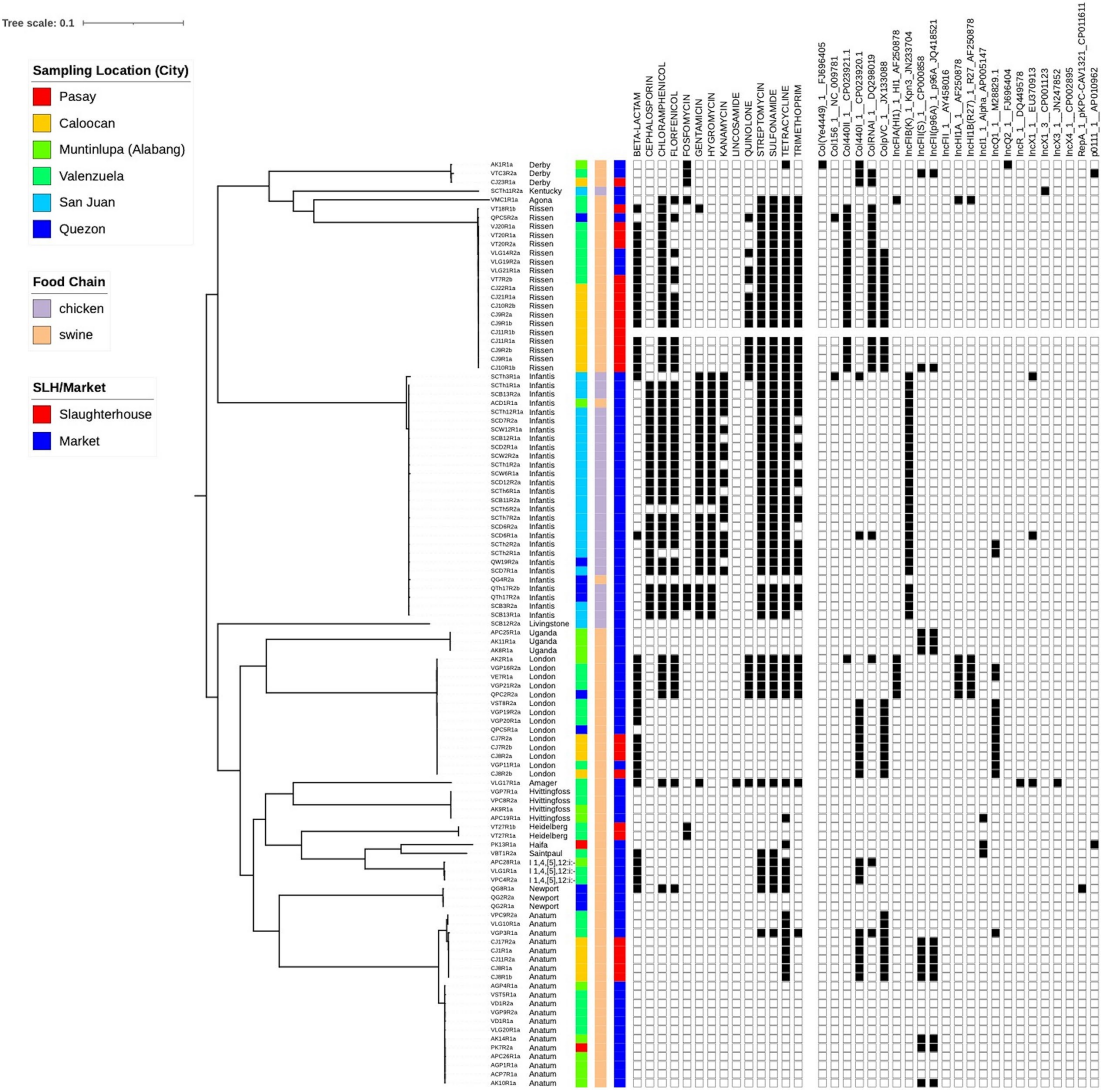
Phylogenetic tree of study isolates (*N* = 105). A core genome analysis was performed to identify genomic regions common to all strains. This was used as input to RaxML to build a maximum likelihood tree. Sampling location, food chain and source type (slaughterhouse/market) are visualized together with phenotypic resistance and the presence of plasmids.

In total, 105 isolates for the study were sourced from six different locations around Metro Manila (Supplementary Figure S1; Supplementary Table S1). These were collected from both markets (74.3%) and abattoirs (25.7%), and from both chicken (26.7%) and swine (73.3%) (Table 1). Phylogenetic reconstruction using a core genome alignment revealed distinct clades which correlated perfectly with the serovars (N = 18) derived from the sequence data (Figure 1). Most of the isolates belonged to the Infantis (*n* = 28, 26.7%), Anatum (20, 19.1%), Rissen (19, 18.1%), and London (14, 13.3%) serovar classes (Table 2).

**Table 1:** Sample characteristics.

Characteristic	*N*	%
Year of collection		
2018	33	31.4
2019	44	41.9
2022	28	26.7
Location*		
Valenzuela	33	31.4
San Juan	25	23.8
Caloocan	20	19.1
Muntinlupa	15	14.3
Quezon	10	9.5
Pasay	2	1.9
Source		
Market	78	74.3
Abattoir	27	25.7
Food chain		
Swine	77	73.3
Chicken	28	26.7
Meat cuts		
Chicken		
Breast	6	5.7
Drumstick	6	5.7
Thigh	12	11.4
Wing	4	3.8
Swine		
Tongue	2	1.9
Meatloaf	1	1.0
Ground	14	13.3
Jejunum	21	20.0
Shoulder	9	8.6
Chorizo	7	6.7
Cubed	1	1.0
Chop	12	11.4
Neck	1	1.0
Cured	3	2.9
Tonsils	6	5.7

^*^All markets, except from abattoirs in Valenzuela (*N* = 7) and Caloocan (*N* = 20).

**Table 2:** Serovars identified.

Serovar	Chicken *N* = 28	Swine *N* = 75	Total *N* = 105	%
Infantis	26	2	28	26.7
Anatum	0	20	20	19.1
Rissen	0	19	19	18.1
London	0	14	14	13.3
Hvittingfoss	0	4	4	3.8
Derby	0	3	3	2.9
I 1,4,[5],12:i:-	0	3	3	2.9
Newport	0	3	3	2.9
Uganda	0	3	3	2.9
Heidelberg	0	2	2	1.9
Agona	0	1	1	<1.0
Amager	0	1	1	<1.0
Haifa	0	0	1	<1.0
Kentucky	1	0	1	<1.0
Livingstone	1	1	1	<1.0
Saintpaul	0	1	1	<1.0

Phenotypic analysis of susceptibility to 15 antimicrobial drugs using VITEK® 2 Compact 60 ID/AST System found that 65% of isolates were resistant to at least one drug, with 57% categorized as extended-spectrum β-lactamases (ESBL) and 37% categorized as MDR. Out of the total 105 strains tested, 63% (66/105) were resistant to ampicillin, 78% (82/105) had intermediate to ciprofloxacin, a quinolone, while 23% (24/105) were resistant. No intermediate resistance to any cephalosporin antimicrobials was detected. Resistance was only observed in cefazolin and ceftriaxone, with 24% (25/105) resistant isolates. For the remaining antibiotics, the resistance percentages were as follows: 31% (33/105) nitrofurantoin, 27% (28/105) gentamicin, 26% (27/105) tobramycin, and 23% (24/105) sulbactam. The isolates were all susceptible to aztreonam, carbapenem, amikacin, and tigecycline (Table 3). The sequences were scanned for AMR loci, revealing the presence of 35 genes and 12 plasmids conferring resistance to 14 different drugs. Most of the isolates (67/105; 64%) exhibited AMR genes against tetracycline (*tetABDM*), followed by 56% of isolates with sulfonamide (*sul1, sul2, sul3*) and streptomycin (*aadA1*, *aadA2*, *aph(6)-Id*, and *aph(3′)-Ib*) AMR genes. In contrast with the phenotypic susceptibility test, there are 56 isolates with trimethoprim-sulfamethoxazole genes (*sul1, sul2, sul3, dfrA1, dfrA12*, and *dfrA14*) but only 9.5% (10/105) of samples exhibited resistance to the antimicrobials. Consistent with the high resistance observed against ampicillin and ciprofloxacin, several isolates have AMR genes and plasmids promoting β-lactam (*bla*_EC-18_, *bla*_CTX-M-65_, *bla*_TEM-1_, *bla*_TEM-150_, and *bla*_TEM-176_) and quinolone resistance (*qnrS1* and *qnrB2*) (Table 3).

**Table 3:** AMR and linked genes and plasmids.

Drug	*N* (%) phenotypic resistant*	Genes	Plasmids
AMP	63.0	*bla*_EC-18_ *bla*_CTX-M-65_ *bla*_TEM-1_ *bla*_TEM-150_ *bla*_TEM-176_	*Col156_1* *Col440I_1* *Col440II_1* *IncFIA(HI1)_1_HI1* *IncFIB(K)_1_Kpn3* *IncFII(p96A)_1_p96A* *IncFII(S)_1* *IncFII_1* *IncFII_1_pSFO* *IncHI1A_1* *IncHI1B(R27)_1_R27* *IncQ1_1* *IncQ2_1* *IncI1_1_Alpha* *IncR_1* *IncX1_1* *IncX1_3* *IncX3_1* *IncX4_1* *p0111_1*
AMS	23.0		
TZP	0		
CZN	24.0	*bla*_EC-18_ *bla*_CTX-M-65_ *bla*_TEM-1_ *bla*_TEM-150_ *bla*_TEM-176_	*IncFIA(HI1)_1_HI1* *IncFIB(K)_1_Kpn3* *IncHI1A_1* *IncHI1B(R27)_1_R27* *IncQ1_1* *IncQ2_1* *IncI1_1_Alpha* *IncR_1* *IncX1_1* *IncX1_3* *IncX3_1* *IncX4_1* *p0111_1* *RepA_1_pKPC-CAV1321*
CTR	24.0		
CEF	0		
NFN	31.0	-	-
GEN	27.0	*aadA1* *aph(4)-Ia* *aac(3)-Iva* *aph(3′)-Ia* *aadA2* *aph(6)-Id* *aph(3″)-Ib* *aac(3)-Iid* *aac(3)-Iie*	*IncFIA(HI1)_1_HI1* *IncFIB(K)_1_Kpn3* *IncHI1A_1* *IncHI1B(R27)_1_R27* *IncQ1_1* *IncQ2_1* *IncI1_1_Alpha* *IncR_1* *IncX1_1* *IncX1_3* *IncX3_1* *IncX4_1* *p0111_1*
SXT	27.0	*sul1* *dfrA14* *dfrA12* *sul2* *sul3* *dfrA1*	-
TOB	26.0	*aadA1* *aph(4)-Ia* *aac(3)-Iva* *aph(3′)-Ia* *aadA2* *aph(6)-Id* *aph(3″)-Ib* *aac(3)-Iid* *aac(3)-Iie*	*IncFIA(HI1)_1_HI1* *IncFIB(K)_1_Kpn3* *IncHI1A_1* *IncHI1B(R27)_1_R27* *IncQ1_1* *IncQ2_1* *IncI1_1_Alpha* *IncR_1* *IncX1_1* *IncX1_3* *IncX3_1* *IncX4_1* *p0111_1*
CIP	23.0	*qnrS1* *qnrB2*	*IncFIB(K)_1_Kpn3* *IncQ1_1* *IncQ2_1* *IncI1_1_Alpha* *IncR_1* *IncX1_1* *IncX1_3* *IncX3_1* *IncX4_1*
AZT	0	*bla*_EC-18_ *bla*_CTX-M-65_ *bla*_TEM-1_ *bla*_TEM-150_ *bla*_TEM-176_	*Col156_1* *Col440I_1* *Col440II_1* *IncFIA(HI1)_1_HI1* *IncFIB(K)_1_Kpn3* *IncFII(p96A)_1_p96A* *IncFII(S)_1* *IncFII_1* *IncFII_1_pSFO* *IncHI1A_1* *IncHI1B(R27)_1_R27* *IncQ1_1* *IncQ2_1* *IncI1_1_Alpha* *IncR_1* *IncX1_1* *IncX1_3* *IncX3_1* *IncX4_1* *p0111_1*
ETP	0	-	*RepA_1_pKPC-CAV1321*
MEM	0	-	
AMK	0	*aadA1* *aph(4)-Ia* *aac(3)-Iva* *aph(3′)-Ia* *aadA2* *aph(6)-Id* *aph(3″)-Ib* *aac(3)-Iid* *aac(3)-Iie*	*IncFIA(HI1)_1_HI1* *IncFIB(K)_1_Kpn3* *IncHI1A_1* *IncHI1B(R27)_1_R27* *IncQ1_1* *IncQ2_1* *IncI1_1_Alpha* *IncR_1* *IncX1_1* *IncX1_3* *IncX3_1* *IncX4_1* *p0111_1* *RepA_1_pKPC-CAV1321*
TGC	0	*tet(A)* *tet(M)* *tet(B)* *tet(D)* *tet(J)*	-

^*^VITEK®; AMP, ampicillin; NFN, nitrofurantoin; GEN, gentamicin; SXT, trimethoprim/sulfamethoxazole; TOB, tobramycin; CZN, cefazolin; CTR, ceftriaxone; CIP, ciprofloxacin; AMS, ampicillin/sulbactam; TZP, piperacillin/tazobactam; CEF, cefepime; AZT, aztreonam; ETP, ertapenem; MEM, meropenem; AMK, amikacin; TGC, tigecycline.

### 3.2 *Salmonella* serovars and AMR

**Figure 2:**
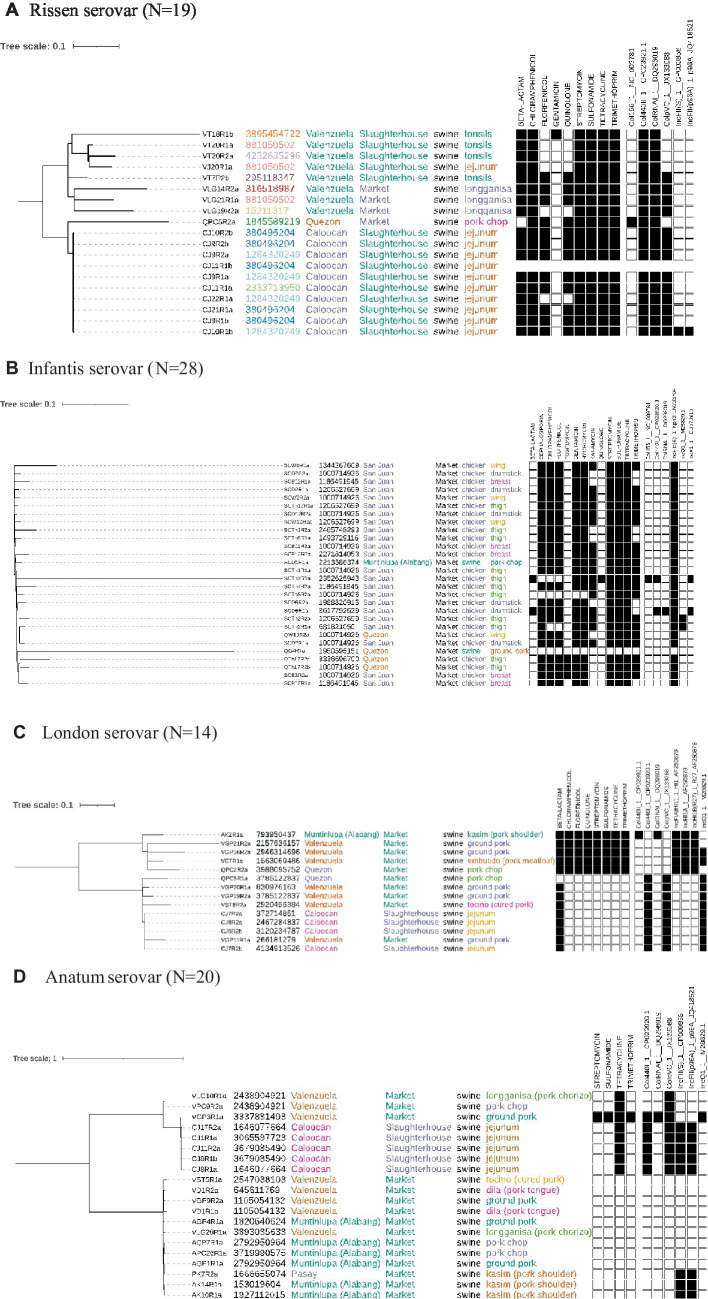
Phylogenetic trees built using on the core genome of isolates across different serovars. Annotated are the sequence IDs, cgMLST ST, source location, type, animal and meat cut. Presence (black square) or absence (white square) of genes conferring resistance to drugs and presence of plasmids are also indicated. **(A)** Rissen serovar (*N* = 19). **(B)** Infantis serovar (*N* = 28). **(C)** London serovar (*N* = 14). **(D)** Anatum serovar (*N* = 20).

Genomic diversity existed within the isolates, but a clonal group is evident from both the phylogenetic tree and the presence of multiple isolates from the same cgMLST ST, all sourced from the same location and meat type. Resistance was common with isolate genomes containing genes that confer resistance to 9 drug types, including some isolates potentially simultaneously resistant to 8 different drugs. Plasmids were also found in all isolates, with two out of six being present across all (Figure 2A).

The Infantis serovar were all sourced from markets, and almost exclusively found in chicken (26/28). Some isolates sourced from different locations had the same cgMLST ST but significant genomic diversity existed preventing close clusters on the phylogenetic tree. AMR was also common in this serovar with genes conferring resistance to 13 different drug groups being found with some isolates presenting resistance to 11 different drugs. Six different plasmids were found with the IncFIB(K) type being present in all samples except one (Figure 2B).

The London serovar was found only in swine and was sourced from both markets and slaughterhouses in four different locations. While some isolates clustered closely on the phylogenetic tree, every isolate had a unique cgMLST ST indicating a significant amount of diversity. Genes conferring resistance to 8 different drugs were found with one clade containing all 8 AMR genes, while the other clade displayed very little resistance with all isolates possessing, at most, one resistance gene. This may be reflective of the different plasmid content between the clades with 8 different plasmids found in total (Figure 2C).

The Anatum serovar was sourced in four different locations and exclusively found in swine from both markets and abattoirs. The phylogenetic tree formed two main clades, and all isolates had different cgMLST STs. AMR loci linked to four different drugs were found, with one isolate presenting all resistance genes. However, one large clade did not present any resistance genes. Six different plasmids were found, including in the clade without resistance genes (Figure 2D). There was evidence of high similarity of samples across different swine markets (Valenzuela, Muntinlupa, Pasay) suggestive of transmission within abattoirs or farms.

### 3.3 Virulence genes

**Figure 3:**
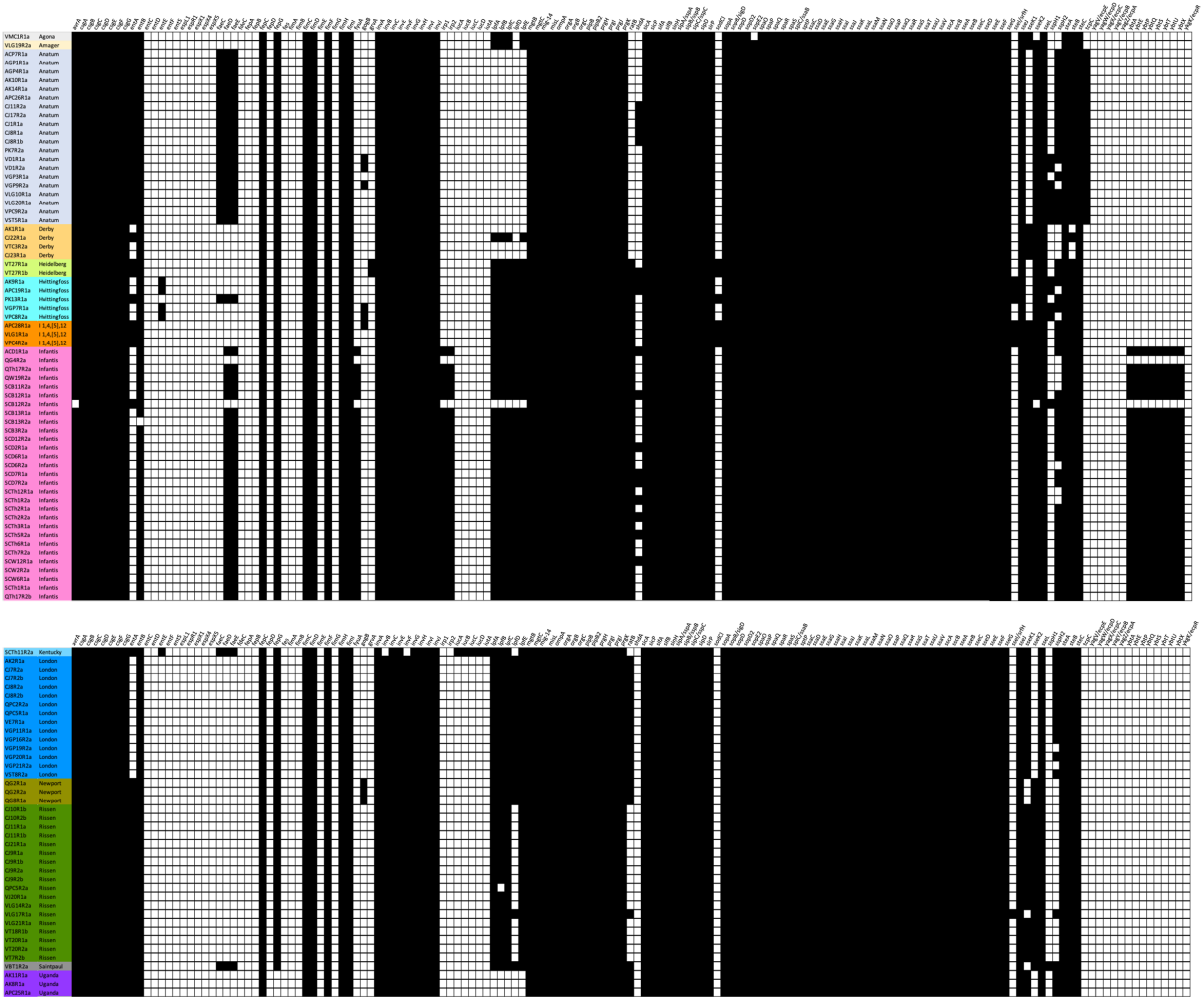
Presence (black square) or absence (white square) of genes conferring virulence characteristics of the isolates. Virulence genes were mined using ABRicate software (v1.0.1; minimum %ID = 80, min % coverage = 80; VFDB database, as of April 4, 2023).

The presence of 155 known virulence genes was detected across the 105 isolates (Supplementary Table S1). More than half of the genes (87/155; 56.12%) were found in all isolates; while a minority (24/155; 15.48%) were absent in all isolates. For the other (non-fixed) 42 virulence genes, some were serovar-specific (Figure 3). The most frequent serovar, Infantis, had the highest number of virulence genes (median number: Infantis 116; vs. other serovars 102; Wilcoxon *p* < 10^−13^). Excluding one outlier, 11 virulence genes were found exclusively in the Infantis serovar (*fyuA, irp1, irp2, ybtA, ybtE, ybtP, ybtQ, ybtS, ybtT, ybtU*, and *ybtX*). Similarly, the aerobactin biosynthesis *iucA, iucB, iucC, iucD*, and *iutA* virulence genes were exclusive to the single Kentucky serovar. The fatty acyl-CoA dehydrogenase (*faeD* and *faeE*) genes were found exclusively in 5 serovars–Anatum (20), Haifa (1), Infantis (27), Kentucky (1), Saintpaul (1).

### 3.4 Genetic similarities within serovars

**Figure 4:**
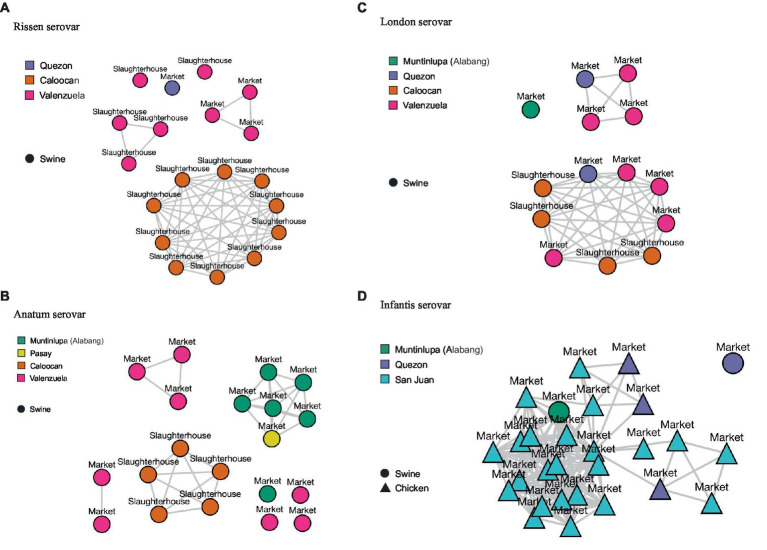
Single-linkage clusters reflecting single-nucleotide polymorphisms (SNPs) at (distance: < 20) within serovars. Sampling sites (market or slaughterhouse), sampling locations (cities), and sample matrices (swine or chicken) are also indicated. **(A)** Rissen serovar. **(B)** Anatum serovar. **(C)** London serovar. **(D)** Infantis serovar.

For the four most isolated serovars, isolates with less than 20 SNPs have been plotted in single-linkage clusters reflecting sampling sites, cities, and matrices in Figure 4.

For the Rissen serovar, isolates sourced from Caloocan slaughterhouse congregated in one cluster, while isolates from Valenzuela slaughterhouse and markets form multiple clusters (Figure 4A). For Anatum serovar, clustering can be observed per sampling site and city, apart from market isolates from Pasay and one isolate from Muntinlupa which have clustered together. Valenzuela Anatum isolates, on the other hand, formed multiple clusters (Figure 4B). Isolates under the London serovar from one slaughterhouse and three markets, all from three cities, are found to congregate in one major cluster. Meanwhile, Valenzuela and Quezon isolates form one minor cluster (Figure 4C). Finally, for the Infantis serovar, the isolates which were mostly sourced from chicken market samples and a few swine samples formed one major cluster which branches out into smaller clusters. Several San Juan isolates link to a few Quezon isolates, forming three minor clusters (Figure 4D).

## 4 Discussion

Due to its emergence, transmission, and persistence, AMR continues to be a serious global health problem. This issue is greatly exacerbated internationally through the use of antimicrobials in various food chains, notably in the production of livestock and poultry. It adversely affects food security in low- and middle-income countries such as the Philippines which is a large consumer of hog and poultry products. This study is the first to report on AMR and transmission of *S. enterica* using a WGS of isolates from swine and poultry in the Philippines.

### 4.1 Serovars

The four most dominant serovars found in this study, Infantis, Anatum, Rissen, and London are consistently detected in several institutional and independent studies in different countries and territories such as the European Union [European Food Safety Authority and European Centre for Disease Prevention and Control (EFSA and ECDC), 2022], the United States (Centers for Disease Control and Prevention (CDC), 2013; Bearson, 2021), and China (Liu et al., 2020; Tang et al., 2022, 2023), to mention a few. In this study, the most dominant is *S.* Infantis which is one of the increasingly emerging and spreading serovars globally (Alvarez et al., 2023) and is widely linked to human salmonellosis (Montone et al., 2023). In our study, this serovar was associated with raw chicken meat, consistent with epidemiological data from the EU [European Food Safety Authority and European Centre for Disease Prevention and Control (EFSA and ECDC), 2022] and the USA [Centers for Disease Control and Prevention (CDC), 2013]. Due to outbreaks in the EU, it has been recommended that this serovar (among others) be included in surveillance schemes (Ferrari et al., 2019).

From the four most dominant serovars, the most AMR genes have been detected in *S.* Infantis. Likewise, *S.* Infantis harbored the most number of virulence genes. These AMR and virulence determinants would have likely propelled the increased emergence and rapid spread of *S.* Infantis. In a large-scale review by Alvarez et al. (2023) which included 3,725 independent studies on *S.* Infantis in different countries, *S.* Infantis isolates collectively exhibited resistance to all antibiotic classes examined, including aminoglycosides, amphenicols, β-lactams, quinolones, sulfonamides, and tetracyclines, among others. They have also exhibited the presence of resistance genes attributed to specific antimicrobials such as *aad, aac, aph, sul, bla, qnr*, and *tet* which are consistent with the results of this study.

The second most dominant serovar was Anatum, which was exclusively isolated from swine samples, consistent with settings such as Latin America (Ferrari et al., 2019). However, previous reviews have revealed that it is associated with beef and seafood, globally, which were not sources considered in our work (Ferrari et al., 2019). The London serovar has been associated with various infections in humans (Yong et al., 2005), animals, and food products (Meunsene et al., 2021). Although it is uncommonly detected and studied among *Salmonella* serovars, London isolates have exhibited potential zoonotic transmission and increasing resistance to antibiotics (Fang et al., 2022). In this study, London isolates were exclusively found in swine samples and only conferred resistance to limited antimicrobial classes. Lastly, the Rissen serovar is currently one of the emerging serovars in various countries worldwide. In a large-scale global review by Elbediwi et al. (2021), it was revealed that the bulk of Rissen isolates (~2/3) were obtained from human samples, more than half of which were asymptomatic individuals. While most non-clinical Rissen isolates in global studies were associated with poultry and porcine samples, several studies have also found the serovar in seafood (Atwill and Jeamsripong, 2021; Lozano-León et al., 2022), and other sources (Prasertsee et al., 2019; Silveira et al., 2019; Nguyen et al., 2021; Sanguankiat et al., 2023).

In the Philippines, *Salmonella* detection studies in non-clinical samples would often be limited to presence-absence tests. Only a few studies would have data on *Salmonella* serovars. One study on retail meats sampled from wet markets in Metro Manila revealed that bovine meat mostly harbored Anatum and Saintpaul, porcine meat mostly harbored Anatum, and poultry meat mostly harbored Kentucky (Santos et al., 2020). However, these identities were only obtained using H typing, and thus are not confirmed. *Salmonella* studies with confirmed serovar identification are limited to clinical samples from the Philippine Department of Health − Antimicrobial Resistance Surveillance Program (DOH-ARSP) of the RITM. In a surveillance study covering blood and stool samples obtained from 17 sentinel sites all over the country from 2013 to 2014, *in silico* genotyping using WGS revealed that the most dominant serovars were *S.* Typhi as well as *S.* Enteritidis and ST34 (I 4, [5],12: i: -) for the non-typhoidal types (Lagrada et al., 2022).

The genetic similarity of the isolates that clustered together can possibly be traced to having the same slaughterhouse or farm source(s), thus harboring the same strains. This can also indicate transmission, as clones of the same strain can be circulating in one sampling site. It is important to note that local farms supply livestock to different slaughterhouses, and these slaughterhouses do not necessarily supply markets within the same city. This means that a farm can send livestock for slaughter to one slaughterhouse, and the slaughtered meat can be supplied to different markets in different cities. Genetically distinct isolates, or those that form multiple clusters, could likely be traced from various slaughterhouses and/or farm sources. Cross-contamination, as apparent in Infantis isolates, could likely be traced to large-scale commercial and backyard farms that rear mixed livestock animals and/or market stalls that sell both pig and chicken meat.

### 4.2 Correlation between AMR and antimicrobial use

*Salmonella* has been found to have intrinsic resistance to β-lactams, macrolides (except azithromycin), lincosamides, glycopeptides, and fusidane (Stock and Wiedemann, 2000). The genetic determinants of these resistances are usually chromosome-encoded or because of functional and/or structural characteristics as opposed to plasmid-borne resistances that can be acquired via horizontal gene transfer. For example, macrolides, a family of antimicrobials that have a characteristic macrocyclic lactone ring structure, have difficulty traversing the Gram-negative cell wall, specifically through the polar and negatively charged outer membrane, consequent to their affinity to efflux pumps that actively transport them out of the cell (McDermott et al., 2003; Myers and Clark, 2021). Another example of this would be their intrinsic resistance to β-lactam antibiotics, such as penicillin which is the highest conferred phenotypic resistance (60/105 isolates) in this study. β-lactam antibiotics, as aptly named, would have a characteristic β-lactam ring, which, similar to macrolides, would have restricted access to the outer membrane. In addition, *S. enterica* would also have a chromosomally-encoded or plasmid-borne *ampC* gene that codes for β-lactamases–enzymes that can hydrolyze β-lactams (Narendrakumar et al., 2023).

The intrinsic resistance of *Salmonella* complicates treatment regimens and raises the need for other treatment options. As this is the case, other drugs of choice are usually prescribed for both typhoidal and non-typhoidal infections such as fluoroquinolones or cephalosporins (Shane et al., 2017; Tack et al., 2020). While these drugs still exhibit efficacy against *Salmonella*, there is a marked increase in the occurrence of intermediate and resistant phenotypes, as exhibited in this study. Alarmingly, there have also been many reports of emerging fluoroquinolone and cephalosporin resistance from various continents. In Russia, fluoroquinolone- and cephalosporin-resistant *Salmonella* strains have been isolated from raw poultry products as well as ready-to-eat chicken products (Egorova et al., 2021). A study in Ghana by Dekker et al. (2018) reported 63% fluoroquinolone resistance in *S. enterica* isolated from local and imported meat. In addition, this resistance is mostly conferred by *qnrB2* resistance plasmids, which means that these resistance determinants can be transferable.

In this study, a significant number of isolates had transferable resistance plasmids such as the *Col* plasmid family that facilitate resistance to glycopeptides (vancomycin), polymyxin (colistin), β-lactam antibiotics (e.g., penicillin and ampicillin), as well as quinolones (ciprofloxacin) (McMillan et al., 2020). The isolates also harbor *Inc* plasmids that confer resistance against aminoglycosides, β-lactams, and fluoroquinolones (Hiley et al., 2021). The modes of action of these resistance genes are primarily related to the production of efflux pumps that actively expel antimicrobial agents from the cell. In addition, several genes code for enzymes that promote the inactivation of antimicrobial agents, either by hydrolysis or through the production of inactivated forms of the drug (Helinski, 2022).

The increasing trend of resistance to fluoroquinolones can be traced to the growth of drug sales, especially for use in livestock settings. A study by Yin et al. (2022) was able to establish a link between a 41.67% increase in fluoroquinolone sales and a 5% increase in the prevalence of quinolone-resistant *Salmonella* in retail meat from 2013 to 2018. In 2020, the estimated use of antimicrobials for the rearing of cattle, sheep, poultry, and swine worldwide was 99,502 tons, with the highest usage recorded in Asia at 67% (66,666 tons). This is particularly important since the volume of imported meat and meat products in the Philippines has ballooned to 16% in 2022, with imports coming in from Brazil, Australia, Germany, India, Italy, and the USA, among others (Bureau of Animal Industry, 2022). Incidentally, these countries are considered antimicrobial use hotspots in 2020 and are projected to remain so come 2050 (Mulchandani et al., 2023). The high consumption of antimicrobials in these hotspot countries could be likely linked to their intensive farming practices to meet local and export demands.

The same literature estimated the global use of tetracycline to be 33,305 tons, making it the most consumed antimicrobial in the world, with the second highest antimicrobial being penicillin, at around 15,000 tons–only half as much as tetracycline. This trend could be correlated with the detection of tetracycline resistance genes in 64% of the isolates. Sulfonamides and aminoglycosides have ranked fourth and sixth, respectively, in global consumption. Resistance genes against these antimicrobials were also detected in 56% of isolates.

### 4.3 Virulence determinants

A total of 155 virulence genes were detected in 105 isolates, 42 of which are serovar-specific. The virulence-associated determinants are grouped into six categories: fimbriae adherence determinants, element-uptake determinants, secretion system, protein synthesis, colonization, and survival against the host immune system.

Fimbriae function as an adhesion factor necessary for bacterial colonization and infection. The prevalence of *csg* and *fim* genes are conserved adhesion and infection factors in *Enterobacteriaceae*. Two *csg* operons, *csg*BAC, and *csg*DEFG, encode for curli fimbriae and mediate binding to tissue matrices, while *fim* genes that encode hair-like appendages called type 1 fimbriae (Römling et al., 1998; Zeiner et al., 2012). These two gene clusters were present in all 105 isolates in this study as they facilitate adhesion and binding to eukaryotic blood and tissue matrices. While *lpf*ACE and *fae*DE were serovar-specific genes determined in the sequence analysis, both were present in Haifa, Kentucky, Saintpaul, and Infantis serovars. These genes are unique for long polar fimbriae and fimbrial adhesin production, respectively. The homologous interchange of horizontally transmitted segments may have allowed the deletion of serovar-specific genes to spread to other *Salmonella* serovars. In particular, the *lpf* operon was initially present in a lineage ancestor of *Salmonella* and has been found to be deleted in several lineages of the genus, contributing to its diverse phylogenetic distribution (Bäumler et al., 1997). Interestingly, most jejunum samples in this study acquired these *lpf* genes since *lpf*-mediated adhesion targeted the alimentary tract such as animal ileum (Bäumler and Heffron, 1995), induced biofilm formation (Ledeboer et al., 2006) and promoted long-term intestinal persistence (Weening et al., 2005), indicating a function-mediated gene deletion and acquisition.

Genes associated with type III secretion system (T3SS) encoded by *Salmonella* pathogenicity islands (SPI) are also predominantly present among all the isolates. Present genes associated with SPI-1 include the *inv*/*spa* and *prg* genes that aid in the invasion and infection of *Salmonella*. The genes encoding for SP 1–2 regulation, and production of its chaperone proteins, effector proteins, and T3SS2 apparatus were also detected. However, the T3SS *esp* genes involved in the regulation and transport of proteins in the host cells during the invasion were absent in all isolates. The lack of genes and operons for the synthesis and regulation of extracellular proteins was also evident.

Other functional sets of genes that are present were responsible for the uptake and transport of organic compounds such as magnesium and iron. All 105 isolates have genes for the uptake of magnesium from the environment. Interestingly, only two serovars possess genes for iron transport and synthesis and encode for a different type of siderophore. The single Kentucky serovar isolate from a chicken sample had genes that encode for aerophore, while most Infantis isolates (27/28) possess genes associated with the uptake, transport, and biosynthesis of yersiniabactin. Iron uptake is important for host-pathogen interactions that are often overlooked in non-typhoidal *Salmonella*. The presence of *Yersinia* high pathogenicity island (HPI) in the Infantis isolates may also promote infection (Fetherston et al., 1996; Oelschlaeger et al., 2003), suppress host immune response (Autenrieth et al., 1991; Gehring et al., 1998) and increase the fitness and persistence in the environment (Oelschlaeger et al., 2003).

The expression of genes associated with pathogenicity islands 1 and 2 as well as fimbriae production among all isolates suggest the potential harm and disease development in humans. The genome sequences in this study served as a basis for traceback investigations to animal and food products as the possible cause of human salmonellosis. A comparative analysis of the SPIs, adhesin molecules, secretion systems, virulence plasmid, and epidemiological characteristics could elucidate the role of food-borne infections in humans.

## 5 Conclusion

Through the application of next-generation sequencing, we found resistance and virulence determinants that contribute to the persistence of *S. enterica* in the poultry and swine food chains in the Philippines. Our study of 105 *S. enterica* isolates provides proof of principle that WGS approaches can decipher the complex AMR and virulence patterns and shows that sequencing should be implemented by meat inspection networks to augment the existing presence−absence detection tests as acceptability, safety, and quality criteria. In addition, WGS gave insights into strain clustering and evidence of infection cross-contamination. Indeed, there are large-scale follow-up studies to assess AMR and virulence diversity and capture transmission, and the current work provides a baseline set of data. Ultimately, the genetic insights from this study and similar works, especially with additional number of samples and isolates could lead to enhanced diagnostics for disease management and control across the entire food chain, including farm, abattoir, and market settings. New genetic markers revealed should be integrated into current testing routines and may replace those currently used to give a more accurate picture of the strains and their AMR profiles. More isolates must be obtained in order to more clearly and accurately visualize the current and possibly predict future trends relating to *S. enterica* transmission, AMR, and virulence. The identification of AMR and transmission chains from farm to fork will assist surveillance and clinical decision-making, thereby improving the food security and health of both humans and animals.

## Data availability statement

The original contributions presented in the study are included in the article/Supplementary materials, further inquiries can be directed to the corresponding authors.

## Ethics statement

The animal study was approved by National Meat Inspection Service, Philippines. The study was conducted in accordance with the local legislation and institutional requirements.

## Author contributions

JM: Data curation, Formal analysis, Investigation, Writing – original draft, Writing – review & editing. VM: Data curation, Formal analysis, Investigation, Writing – original draft. AC: Data curation, Investigation, Writing – original draft. SC: Conceptualization, Writing – review & editing. JH: Conceptualization, Methodology, Writing – review & editing. MH: Conceptualization, Methodology, Writing – original draft. JP: Data curation, Formal analysis, Methodology, Software, Writing – original draft. TC: Conceptualization, Funding acquisition, Methodology, Project administration, Resources, Supervision, Validation, Visualization, Writing – original draft, Writing – review & editing. WR: Conceptualization, Funding acquisition, Methodology, Project administration, Resources, Supervision, Validation, Visualization, Writing – original draft, Writing – review & editing.

## Footnotes

^1^ https://github.com/tseemann/snippy

^2^ https://jodyphelan.github.io/tgv/
