# Supplementary material for: Genomic analysis of Salmonella enterica from Metropolitan Manila abattoirs and markets reveals insights into circulating virulence and antimicrobial resistance genotypes
Source: Front Microbiol. 2024 Jan 19;14:1304283. doi: 10.3389/fmicb.2023.1304283 (PMC10835624; doi:10.3389/fmicb.2023.1304283)
Supplement: Supplementary file 1 [file Data_Sheet_1.PDF]

Table S1. Metadata for the isolates

Sample No.	Sample ID	Location	Source	Food Chain	Year	Serovar	Accession
1	APC19R1a	Muntinlupa	Market	swine	2019	Hvittingfoss	ERR12302296
2	APC25R1a	Muntinlupa	Market	swine	2019	Uganda	ERR12302320
3	APC28R1a	Muntinlupa	Market	swine	2019	I 1,4,[5],12	ERR12302276
4	AGP1R1a	Muntinlupa	Market	swine	2019	Anatum	ERR12302259
5	AGP4R1a	Muntinlupa	Market	swine	2019	Anatum	ERR12302258
6	AK1R1a	Muntinlupa	Market	swine	2019	Derby	ERR12302281
7	AK2R1a	Muntinlupa	Market	swine	2019	London	ERR12302265
8	AK8R1a	Muntinlupa	Market	swine	2019	Uganda	ERR12302314
9	AK9R1a	Muntinlupa	Market	swine	2019	Hvittingfoss	ERR12302302
10	AK10R1a	Muntinlupa	Market	swine	2019	Anatum	ERR12302287
11	AK11R1a	Muntinlupa	Market	swine	2019	Uganda	ERR12302289
12	AK14R1a	Muntinlupa	Market	swine	2019	Anatum	ERR12302274
13	ACP7R1a	Muntinlupa	Market	swine	2019	Anatum	ERR12302260
14	ACD1R1a	Muntinlupa	Market	swine	2019	Infantis	ERR12302352
15	VT7R2b	Valenzuela	Abattoir	swine	2018	Rissen	ERR12302288
16	VT20R1a	Valenzuela	Abattoir	swine	2018	Rissen	ERR12302315
17	VT20R2a	Valenzuela	Abattoir	swine	2018	Rissen	ERR12302349
18	VT18R1b	Valenzuela	Abattoir	swine	2018	Rissen	ERR12302300
19	VT27R1a	Valenzuela	Abattoir	swine	2018	Heidelberg	ERR12302322
20	VT27R1b	Valenzuela	Abattoir	swine	2018	Heidelberg	ERR12302317
21	VJ20R1a	Valenzuela	Abattoir	swine	2018	Rissen	ERR12302304
22	VD1R1a	Valenzuela	Market	swine	2018	Anatum	ERR12302278
23	VD1R2a	Valenzuela	Market	swine	2018	Anatum	ERR12302341
24	VE7R1a	Valenzuela	Market	swine	2018	London	ERR12302344
25	VMC1R1a	Valenzuela	Market	swine	2018	Agona	ERR12302311
26	VBT1R2a	Valenzuela	Market	swine	2018	Saintpaul	ERR12302293
27	VGP3R1a	Valenzuela	Market	swine	2018	Anatum	ERR12302301
28	VGP7R1a	Valenzuela	Market	swine	2018	Hvittingfoss	ERR12302285

29	VGP9R2a	Valenzuela	Market	swine	2018	Anatum	ERR12302307
30	VGP11R1a	Valenzuela	Market	swine	2018	London	ERR12302332
31	VGP16R2a	Valenzuela	Market	swine	2018	London	ERR12302271
32	VGP19R2a	Valenzuela	Market	swine	2018	London	ERR12302319
33	VGP20R1a	Valenzuela	Market	swine	2018	London	ERR12302292
34	VGP21R2a	Valenzuela	Market	swine	2018	London	ERR12302313
35	VLG1R1a	Valenzuela	Market	swine	2018	I 1,4,[5],12	ERR12302270
36	VLG10R1a	Valenzuela	Market	swine	2018	Anatum	ERR12302327
37	VLG14R2a	Valenzuela	Market	swine	2018	Rissen	ERR12302275
38	VLG17R1a	Valenzuela	Market	swine	2018	Amager	ERR12302264
39	VLG19R2a	Valenzuela	Market	swine	2018	Rissen	ERR12302277
40	VLG20R1a	Valenzuela	Market	swine	2018	Anatum	ERR12302268
41	VLG21R1a	Valenzuela	Market	swine	2018	Rissen	ERR12302308
42	VST5R1a	Valenzuela	Market	swine	2018	Anatum	ERR12302294
43	VST8R2a	Valenzuela	Market	swine	2018	London	ERR12302272
44	VTC3R2a	Valenzuela	Market	swine	2018	Derby	ERR12302305
45	VPC4R2a	Valenzuela	Market	swine	2018	I 1,4,[5],12	ERR12302310
46	VPC8R2a	Valenzuela	Market	swine	2018	Hvittingfoss	ERR12302257
47	VPC9R2a	Valenzuela	Market	swine	2018	Anatum	ERR12302273
48	QPC2R2a	Quezon	Market	swine	2019	London	ERR12302279
49	QPC5R1a	Quezon	Market	swine	2019	London	ERR12302254
50	QPC5R2a	Quezon	Market	swine	2019	Rissen	ERR12302297
51	QG2R1a	Quezon	Market	swine	2019	Newport	ERR12302266
52	QG2R2a	Quezon	Market	swine	2019	Newport	ERR12302316
53	QG4R2a	Quezon	Market	swine	2019	Infantis	ERR12302253
54	QG8R1a	Quezon	Market	swine	2019	Newport	ERR12302342
55	PK7R2a	Pasay	Market	swine	2019	Anatum	ERR12302318
56	PK13R1a	Pasay	Market	swine	2019	Haifa	ERR12302354
57	SCD2R1a	San Juan	Market	chicken	2022	Infantis	ERR12302348
58	SCD6R1a	San Juan	Market	chicken	2022	Infantis	ERR12302345
59	SCD6R2a	San Juan	Market	chicken	2022	Infantis	ERR12302331

60	SCD7R1a	San Juan	Market	chicken	2022	Infantis	ERR12302306
61	SCD7R2a	San Juan	Market	chicken	2022	Infantis	ERR12302329
62	SCD12R2a	San Juan	Market	chicken	2022	Infantis	ERR12302291
63	SCB3R2a	San Juan	Market	chicken	2022	Infantis	ERR12302312
64	SCB11R2a	San Juan	Market	chicken	2022	Infantis	ERR12302321
65	SCB12R1a	San Juan	Market	chicken	2022	Infantis	ERR12302261
66	SCB12R2a	San Juan	Market	chicken	2022	Livingstone	ERR12302328
67	SCB13R1a	San Juan	Market	chicken	2022	Infantis	ERR12302262
68	SCB13R2a	San Juan	Market	chicken	2022	Infantis	ERR12302309
69	SCTh1R1a	San Juan	Market	chicken	2022	Infantis	ERR12304007
70	SCTh1R2a	San Juan	Market	chicken	2022	Infantis	ERR12302335
71	SCTh2R1a	San Juan	Market	chicken	2022	Infantis	ERR12302290
72	SCTh2R2a	San Juan	Market	chicken	2022	Infantis	ERR12302350
73	SCTh3R1a	San Juan	Market	chicken	2022	Infantis	ERR12302330
74	SCTh5R2a	San Juan	Market	chicken	2022	Infantis	ERR12302333
75	SCTh6R1a	San Juan	Market	chicken	2022	Infantis	ERR12302353
76	SCTh7R2a	San Juan	Market	chicken	2022	Infantis	ERR12302299
77	SCTh11R2a	San Juan	Market	chicken	2022	Kentucky	ERR12302282
78	SCTh12R1a	San Juan	Market	chicken	2022	Infantis	ERR12302347
79	SCW2R2a	San Juan	Market	chicken	2022	Infantis	ERR12302337
80	SCW6R1a	San Juan	Market	chicken	2022	Infantis	ERR12302280
81	SCW12R1a	San Juan	Market	chicken	2022	Infantis	ERR12302346
82	QW19R2a	Quezon	Market	chicken	2022	Infantis	ERR12302340
83	QTh17R2a	Quezon	Market	chicken	2022	Infantis	ERR12302323
84	QTh17R2b	Quezon	Market	chicken	2022	Infantis	ERR12304006
85	APC26R1a	Muntinlupa	Market	swine	2019	Anatum	ERR12302336
86	CJ1R1a	Caloocan	Abattoir	swine	2019	Anatum	ERR12302343
87	CJ7R2a	Caloocan	Abattoir	swine	2019	London	ERR12302286
88	CJ7R2b	Caloocan	Abattoir	swine	2019	London	ERR12302339
89	CJ8R1a	Caloocan	Abattoir	swine	2019	Anatum	ERR12302263
90	CJ8R1b	Caloocan	Abattoir	swine	2019	Anatum	ERR12302326

91	CJ8R2a	Caloocan	Abattoir	swine	2019	London	ERR12302334
92	CJ8R2b	Caloocan	Abattoir	swine	2019	London	ERR12302325
93	CJ9R1a	Caloocan	Abattoir	swine	2019	Rissen	ERR12302283
94	CJ9R1b	Caloocan	Abattoir	swine	2019	Rissen	ERR12302295
95	CJ9R2a	Caloocan	Abattoir	swine	2019	Rissen	ERR12302267
96	CJ9R2b	Caloocan	Abattoir	swine	2019	Rissen	ERR12302284
97	CJ10R1b	Caloocan	Abattoir	swine	2019	Rissen	ERR12302351
98	CJ10R2b	Caloocan	Abattoir	swine	2019	Rissen	ERR12302338
99	CJ11R1a	Caloocan	Abattoir	swine	2019	Rissen	ERR12302303
100	CJ11R1b	Caloocan	Abattoir	swine	2019	Rissen	ERR12302298
101	CJ11R2a	Caloocan	Abattoir	swine	2019	Anatum	ERR12302256
102	CJ17R2a	Caloocan	Abattoir	swine	2019	Anatum	ERR12302255
103	CJ21R1a	Caloocan	Abattoir	swine	2019	Rissen	ERR12302324
104	CJ22R1a	Caloocan	Abattoir	swine	2019	Derby	ERR12302269
105	CJ23R1a	Caloocan	Abattoir	swine	2019	Derby	ERR12304007

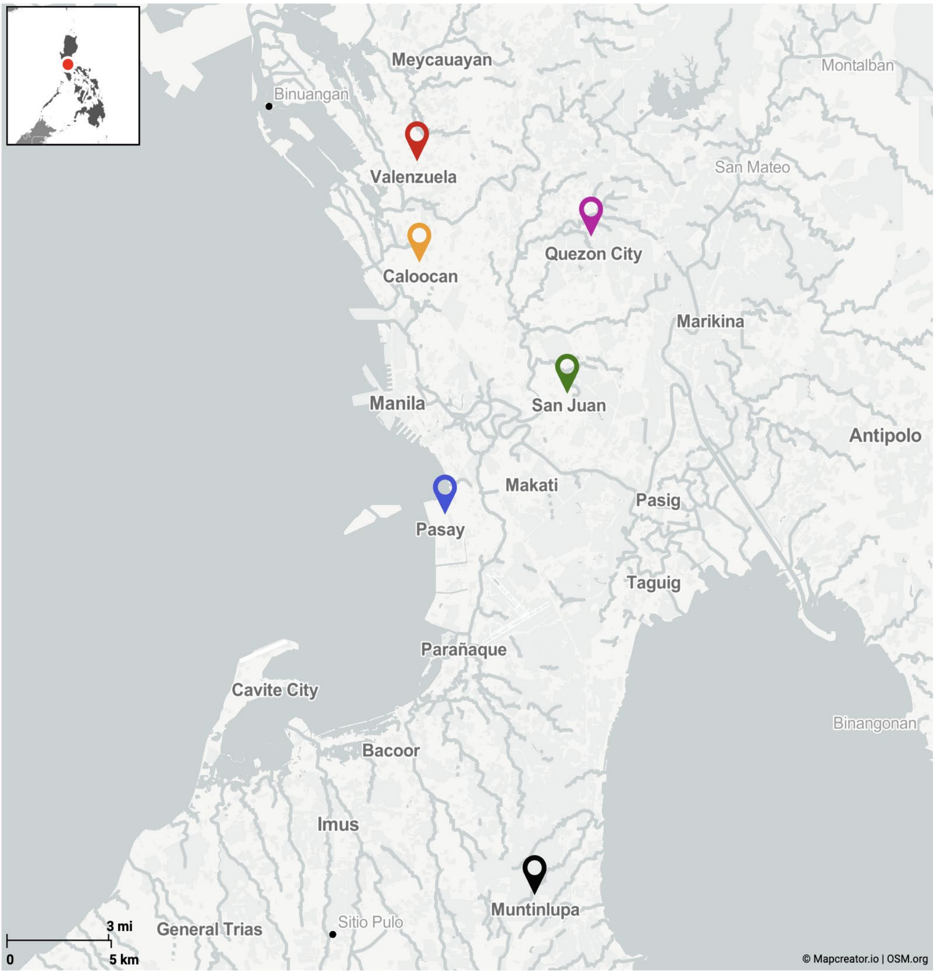

Figure S1. Map of sampling sites in Metro Manila. Samples were collected from eight cities. Samples included in this paper were from Valenzuela, San Juan, Caloocan, Muntinlupa, Quezon, and Pasay.
